# Model-based Analysis of ChIP-Seq (MACS)

**DOI:** 10.1186/gb-2008-9-9-r137

**Published:** 2008-09-17

**Authors:** Yong Zhang, Tao Liu, Clifford A Meyer, Jérôme Eeckhoute, David S Johnson, Bradley E Bernstein, Chad Nusbaum, Richard M Myers, Myles Brown, Wei Li, X Shirley Liu

**Affiliations:** 1 Department of Biostatistics and Computational Biology, Dana-Farber Cancer Institute and Harvard School of Public Health, 44 Binney Street, Boston, MA 02115, USA; 2 Division of Molecular and Cellular Oncology, Department of Medical Oncology, Dana-Farber Cancer Institute and Department of Medicine, Brigham and Women's Hospital and Harvard Medical School, 44 Binney Street, Boston, MA 02115, USA; 3 Gene Security Network, Inc., 2686 Middlefield Road, Redwood City, CA 94063, USA; 4 Molecular Pathology Unit and Center for Cancer Research, Massachusetts General Hospital and Department of Pathology, Harvard Medical School, 13th Street, Charlestown, MA 02129, USA; 5 Broad Institute of Harvard and MIT, 7 Cambridge Center, Cambridge, MA, 02142, USA; 6 Department of Genetics, Stanford University Medical Center, Stanford, CA 94305, USA; 7 Division of Biostatistics, Dan L Duncan Cancer Center, Department of Molecular and Cellular Biology, Baylor College of Medicine, One Baylor Plaza, Houston, TX 77030, USA

## Abstract

We present Model-based Analysis of ChIP-Seq data, MACS, which analyzes data generated by short read sequencers such as Solexa's Genome Analyzer. MACS empirically models the shift size of ChIP-Seq tags, and uses it to improve the spatial resolution of predicted binding sites. MACS also uses a dynamic Poisson distribution to effectively capture local biases in the genome, allowing for more robust predictions. MACS compares favorably to existing ChIP-Seq peak-finding algorithms, and is freely available.

## Abstract 2

MACS performs model-based analysis of ChIP-Seq data generated by short read sequencers.

## Background

The determination of the 'cistrome', the genome-wide set of *in vivo cis*-elements bound by *trans*-factors [1], is necessary to determine the genes that are directly regulated by those *trans*-factors. Chromatin immunoprecipitation (ChIP) [2] coupled with genome tiling microarrays (ChIP-chip) [3,4] and sequencing (ChIP-Seq) [5-8] have become popular techniques to identify cistromes. Although early ChIP-Seq efforts were limited by sequencing throughput and cost [2,9], tremendous progress has been achieved in the past year in the development of next generation massively parallel sequencing. Tens of millions of short tags (25-50 bases) can now be simultaneously sequenced at less than 1% the cost of traditional Sanger sequencing methods. Technologies such as Illumina's Solexa or Applied Biosystems' SOLiD™ have made ChIP-Seq a practical and potentially superior alternative to ChIP-chip [5,8].

While providing several advantages over ChIP-chip, such as less starting material, lower cost, and higher peak resolution, ChIP-Seq also poses challenges (or opportunities) in the analysis of data. First, ChIP-Seq tags represent only the ends of the ChIP fragments, instead of precise protein-DNA binding sites. Although tag strand information and the approximate distance to the precise binding site could help improve peak resolution, a good tag to site distance estimate is often unknown to the user. Second, ChIP-Seq data exhibit regional biases along the genome due to sequencing and mapping biases, chromatin structure and genome copy number variations [10]. These biases could be modeled if matching control samples are sequenced deeply enough. However, among the four recently published ChIP-Seq studies [5-8], one did not have a control sample [5] and only one of the three with control samples systematically used them to guide peak finding [8]. That method requires peaks to contain significantly enriched tags in the ChIP sample relative to the control, although a small ChIP peak region often contains too few control tags to robustly estimate the background biases.

Here, we present Model-based Analysis of ChIP-Seq data, MACS, which addresses these issues and gives robust and high resolution ChIP-Seq peak predictions. We conducted ChIP-Seq of FoxA1 (hepatocyte nuclear factor 3α) in MCF7 cells for comparison with FoxA1 ChIP-chip [1] and identification of features unique to each platform. When applied to three human ChIP-Seq datasets to identify binding sites of FoxA1 in MCF7 cells, NRSF (neuron-restrictive silencer factor) in Jurkat T cells [8], and CTCF (CCCTC-binding factor) in CD4^+ ^T cells [5] (summarized in Table S1 in Additional data file 1), MACS gives results superior to those produced by other published ChIP-Seq peak finding algorithms [8,11,12].

## Results

### Modeling the shift size of ChIP-Seq tags

ChIP-Seq tags represent the ends of fragments in a ChIP-DNA library and are often shifted towards the 3' direction to better represent the precise protein-DNA interaction site. The size of the shift is, however, often unknown to the experimenter. Since ChIP-DNA fragments are equally likely to be sequenced from both ends, the tag density around a true binding site should show a bimodal enrichment pattern, with Watson strand tags enriched upstream of binding and Crick strand tags enriched downstream. MACS takes advantage of this bimodal pattern to empirically model the shifting size to better locate the precise binding sites.

**Figure 1:**
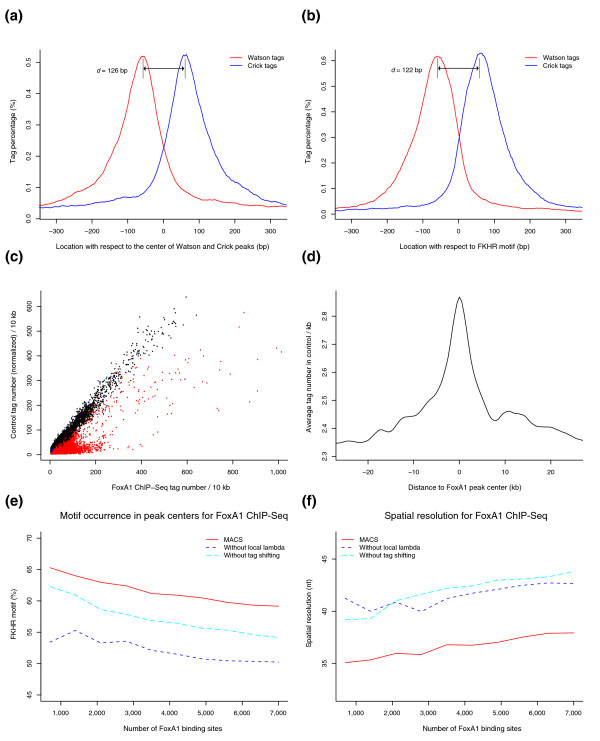
MACS model for FoxA1 ChIP-Seq. **(a,b) **The 5' ends of strand-separated tags from a random sample of 1,000 model peaks, aligned by the center of their Watson and Crick peaks (a) and by the FKHR motif (b). **(c) **The tag count in ChIP versus control in 10 kb windows across the genome. Each dot represents a 10 kb window; red dots are windows containing ChIP peaks and black dots are windows containing control peaks used for FDR calculation. **(d) **Tag density profile in control samples around FoxA1 ChIP-Seq peaks. **(e,f) **MACS improves the motif occurrence in the identified peak centers (e) and the spatial resolution (f) for FoxA1 ChIP-Seq through tag shifting and λ_local_. Peaks are ranked by *p*-value. The motif occurrence is calculated as the percentage of peaks with the FKHR motif within 50 bp of the peak *summit*. The spatial resolution is calculated as the average distance from the *summit *to the nearest FKHR motif. Peaks with no FKHR motif within 150 bp of the peak *summit *are removed from the spatial resolution calculation.

Given a sonication size (*bandwidth*) and a high-confidence fold-enrichment (*mfold*), MACS slides *2bandwidth *windows across the genome to find regions with tags more than *mfold *enriched relative to a random tag genome distribution. MACS randomly samples 1,000 of these high-quality peaks, separates their Watson and Crick tags, and aligns them by the midpoint between their Watson and Crick tag centers (Figure 1a) if the Watson tag center is to the left of the Crick tag center. The distance between the modes of the Watson and Crick peaks in the alignment is defined as '*d*', and MACS shifts all the tags by *d*/*2 *toward the 3' ends to the most likely protein-DNA interaction sites.

When applied to FoxA1 ChIP-Seq, which was sequenced with 3.9 million uniquely mapped tags, MACS estimates the *d *to be only 126 bp (Figure 1a; suggesting a tag shift size of 63 bp), despite a sonication size (*bandwidth*) of around 500 bp and Solexa size-selection of around 200 bp. Since the FKHR motif sequence dictates the precise FoxA1 binding location, the true distribution of *d *could be estimated by aligning the tags by the FKHR motif (122 bp; Figure 1b), which gives a similar result to the MACS model. When applied to NRSF and CTCF ChIP-Seq, MACS also estimates a reasonable *d *solely from the tag distribution: for NRSF ChIP-Seq the MACS model estimated *d *as 96 bp compared to the motif estimate of 70 bp; applied to CTCF ChIP-Seq data the MACS model estimated a *d *of 76 bp compared to the motif estimate of 62 bp.

### Peak detection

For experiments with a control, MACS linearly scales the total control tag count to be the same as the total ChIP tag count. Sometimes the same tag can be sequenced repeatedly, more times than expected from a random genome-wide tag distribution. Such tags might arise from biases during ChIP-DNA amplification and sequencing library preparation, and are likely to add noise to the final peak calls. Therefore, MACS removes duplicate tags in excess of what is warranted by the sequencing depth (binomial distribution *p*-value <10^-5^). For example, for the 3.9 million FoxA1 ChIP-Seq tags, MACS allows each genomic position to contain no more than one tag and removes all the redundancies.

With the current genome coverage of most ChIP-Seq experiments, tag distribution along the genome could be modeled by a Poisson distribution [7]. The advantage of this model is that one parameter, λ_BG_, can capture both the mean and the variance of the distribution. After MACS shifts every tag by *d*/*2*, it slides *2d *windows across the genome to find candidate peaks with a significant tag enrichment (Poisson distribution *p*-value based on λ_BG_, default 10^-5^). Overlapping enriched peaks are merged, and each tag position is extended *d *bases from its center. The location with the highest fragment pileup, hereafter referred to as the *summit*, is predicted as the precise binding location.

In the control samples, we often observe tag distributions with local fluctuations and biases. For example, at the FoxA1 candidate peak locations, tag counts are well correlated between ChIP and control samples (Figure 1c,d). Many possible sources for these biases include local chromatin structure, DNA amplification and sequencing bias, and genome copy number variation. Therefore, instead of using a uniform λ_BG _estimated from the whole genome, MACS uses a dynamic parameter, λ_local_, defined for each candidate peak as:

λ_local _= max(λ_BG_, [λ_1k_,] λ_5k_, λ_10k_)

where λ_1k_, λ_5k _and λ_10k _are λ estimated from the 1 kb, 5 kb or 10 kb window centered at the peak location in the control sample, or the ChIP-Seq sample when a control sample is not available (in which case λ_1k _is not used). λ_local _captures the influence of local biases, and is robust against occasional low tag counts at small local regions. MACS uses λ_local _to calculate the *p*-value of each candidate peak and removes potential false positives due to local biases (that is, peaks significantly under λ_BG_, but not under λ_local_). Candidate peaks with *p*-values below a user-defined threshold *p*-value (default 10^-5^) are called, and the ratio between the ChIP-Seq tag count and λ_local _is reported as the *fold_enrichment*.

For a ChIP-Seq experiment with controls, MACS empirically estimates the false discovery rate (FDR) for each detected peak using the same procedure employed in the previous ChIP-chip peak finders MAT [13] and MA2C [14]. At each *p*-value, MACS uses the same parameters to find ChIP peaks over control and control peaks over ChIP (that is, a sample swap). The empirical FDR is defined as Number of control peaks / Number of ChIP peaks. MACS can also be applied to differential binding between two conditions by treating one of the samples as the control. Since peaks from either sample are likely to be biologically meaningful in this case, we cannot use a sample swap to calculate FDR, and the data quality of each sample needs to be evaluated against a real control.

### Model evaluation

The two key features of MACS are: empirical modeling of '*d*' and tag shifting by *d*/*2 *to putative protein-DNA interaction site; and the use of a dynamic λ_local _to capture local biases in the genome. To evaluate the effectiveness of tag shifting based on the MACS model *d*, we compared the performance of MACS to a similar procedure that uses the original tag positions instead of the shifted tag locations. The effectiveness of the dynamic λ_local _is assessed by comparing MACS to a procedure that uses a uniform λ_BG _from the genome background. Figure 1e,f show that both the detection specificity, measured by the percentage of predicted peaks with a FKHR motif within 50 bp of the peak *summit*, and the spatial resolution, defined as the average distance from the peak *summit *to the nearest FKHR motif, are greatly improved by using tag shifting and the dynamic λ_local_. In addition, FoxA1 is known to cooperatively interact with estrogen receptor in breast cancer cells [1,15]. As evidence for this, we also observed enrichment for estrogen receptor elements (3.1-fold enriched relative to genome motif occurrence) and its half-site (2.7-fold) [15] within the center 300 bp regions of MACS-detected FoxA1 ChIP-Seq peaks.

λ_local _is also effective in capturing the local genomic bias from a ChIP sample alone when a control is not available. To demonstrate this, we applied MACS to FoxA1 ChIP-Seq and control data separately. Using the same parameters, all the control peaks are, in theory, false positives, so the FDR can be empirically estimated as Number of control peaks / Number of ChIP peaks. To identify 7,000 peaks, the FDR for MACS is only 0.4% when a control is available and λ_local _is used. To get 7,000 peaks when a control is not available, the FDR could still remain low at 3.8% if MACS estimates λ_local _from the ChIP sample, whereas it would reach 41.2% if MACS uses a global λ_BG_. This implies that the λ_local _is critical for ChIP-Seq studies when matching control samples are not available [5,9].

### Method comparisons

**Figure 2:**
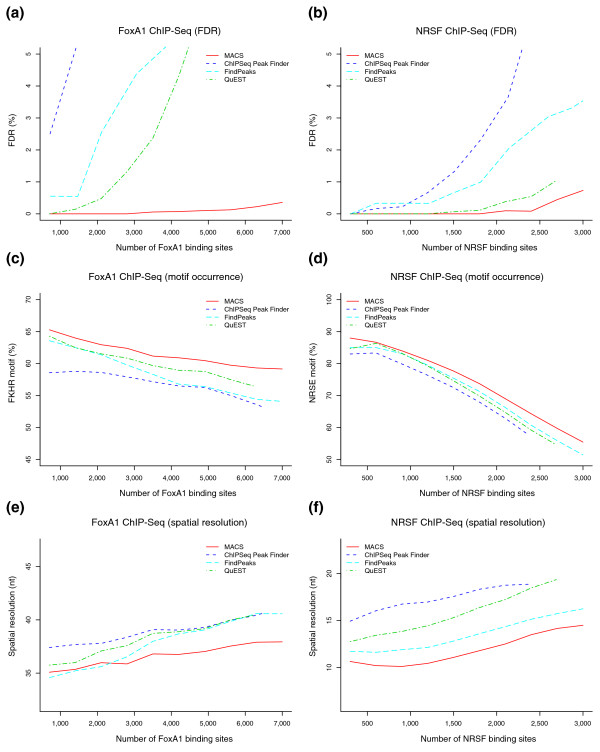
Comparison of MACS with ChIPSeq Peak Finder, FindPeaks and QuEST. **(a-f) **Shown is the FDR for FoxA1 (a) and NRSF (b) ChIP-Seq, motif occurrence within 50 bp of the peak centers for FoxA1 (c) and NRSF (d), and the average distance from the peak center to the nearest motif (peaks with no motif within 150 bp from peak center are removed) for FoxA1 (e) and NRSF (f).

We compared MACS with three other publicly available ChIP-Seq peak finding methods, ChIPSeq Peak Finder [8], FindPeaks [11] and QuEST [12]. To compare their prediction specificity, we swapped the ChIP and control samples, and calculated the FDR of each algorithm as Number of control peaks / Number of ChIP peaks using the same parameters for ChIP and control. For FoxA1 and NRSF ChIP-Seq (an FDR for CTCF is not available due to the lack of control), MACS consistently gave fewer false positives than the other three methods (Figure 2a,b).

Determining the percentage of predicted peaks associated with a motif within 50 bp of the peak center for FoxA1 and NRSF ChIP-Seq, we found MACS to give consistently higher motif occurrences (Figure 2c,d). Evaluating the average distance from peak center to motif, excluding peaks that have no motif within 150 bp of the peak center, we found that MACS predicts peaks with better spatial resolution in most cases (Figure 2e,f). For CTCF, since QuEST does not run on samples without controls, we only compared MACS to ChIPSeq Peak Finder and FindPeaks. Again, MACS gave both higher motif occurrences within 50 bp of the peak center and better spatial resolutions than other methods (Figure S1 in Additional data file 1). In general, MACS not only found more peaks with fewer false positives, but also provided better binding resolution to facilitate downstream motif discovery.

### Comparison of ChIP-Seq to ChIP-chip

**Figure 3:**
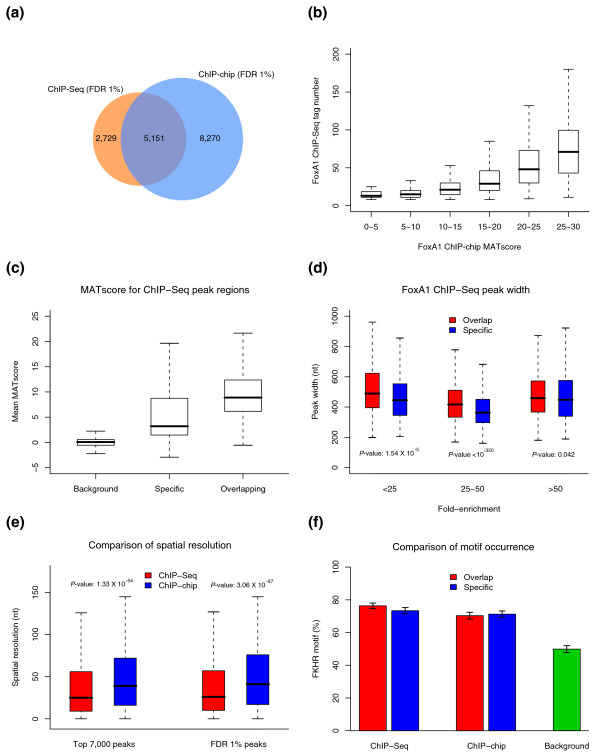
Comparison of FoxA1 ChIP-Seq and ChIP-chip. **(a) **Overlap between the FoxA1 binding sites detected by ChIP-chip (MAT; FDR <1% and fold-enrichment >2) and ChIP-Seq (MACS; FDR <1%). Shown are the numbers of regions detected by both platforms (that is, having at least 1 bp in common) or unique to each platform. **(b) **The distributions of ChIP-Seq tag number and ChIP-chip MATscore [13] for FoxA1 binding sites identified by both platforms. **(c) **MATscore distributions of FoxA1 ChIP-chip at ChIP-Seq/chip overlapping peaks, ChIP-Seq unique peaks, and genome background. For each peak, the mean MATscore for all probes within the 300 bp region centered at the ChIP-Seq peak *summit *is used. Genome background is based on MATscores of all array probes in the FoxA1 ChIP-chip data. **(d) **Width distributions of FoxA1 ChIP-Seq/chip overlapping peaks and ChIP-Seq unique peaks at different fold-enrichments (less than 25, 25 to 50, and larger than 50). **(e) **Spatial resolution for FoxA1 ChIP-chip and ChIP-Seq peaks. The Wilcoxon test was used to calculate the *p*-values for (d) and (e). **(f) **Motif occurrence within the central 200 bp regions for FoxA1 ChIP-Seq/chip overlapping peaks and platform unique peaks. Error bars showing standard deviation were calculated from random sampling of 500 peaks ten times for each category. Background motif occurrences are based on 100,000 randomly selected 200 bp regions in the human genome, excluding regions in genome assembly gaps (containing 'N').

A comparison of FoxA1 ChIP-Seq and ChIP-chip revealed the peak locations to be fairly consistent with each other (Figure 3a). Not surprisingly, the majority of ChIP-Seq peaks under a FDR of 1% (65.4%) were also detected by ChIP-chip (MAT [13] cutoff at FDR <1% and fold-enrichment >2). Among the remaining 34.6% ChIP-Seq unique peaks, 1,045 (13.3%) were not tiled or only partially tiled on the arrays due to the array design. Therefore, only 21.4% of ChIP-Seq peaks are indeed specific to the sequencing platform. Furthermore, ChIP-chip targets with higher fold-enrichments are more likely to be reproducibly detected by ChIP-Seq with a higher tag count (Figure 3b). Meanwhile, although the signals of array probes at the ChIP-Seq specific peak regions are below the peak-calling cutoff, they show moderate signal enrichments that are significantly higher than the genomic background (Wilcoxon *p*-value <10^-320^; Figure 3c). Indeed, 835 out of 1,684 ChIP-Seq specific peaks could also be detected in ChIP-chip, when the less stringent FDR cutoff of 5% is used. Another reason why peaks detected by ChIP-Seq may be undetected by ChIP-chip is that ChIP-Seq specific peaks are usually slightly shorter than similar fold-enrichment peaks found by both ChIP-Seq and ChIP-chip (Figure 3d) and may not be detectable on the array due to insufficient probe coverage. On the other hand, ChIP-chip specific peak regions also have significantly more sequencing tags than the genomic background (Wilcoxon *p*-value <10^-320^; Figure S2 in Additional data file 1), although with current sequencing depth, those regions cannot be called as peaks.

Comparing the difference between ChIP-chip and ChIP-Seq peaks, we find that the average peak width from ChIP-chip is twice as large as that from ChIP-Seq. The average distance from peak *summit *to motif is significantly smaller in ChIP-Seq than ChIP-chip (Figure 3e), demonstrating the superior resolution of ChIP-Seq. Under the same 1% FDR cutoff, the FKHR motif occurrence within the central 200 bp from ChIP-chip or ChIP-Seq specific peaks is comparable with that from the overlapping peaks (Figure 3f). This suggests that most of the platform-specific peaks are genuine binding sites. A comparison between NRSF ChIP-Seq and ChIP-chip (Figure S3 in Additional data file 1) yields similar results, although the overlapping peaks for NRSF are of much better quality than the platform-specific peaks.

## Discussion

ChIP-Seq users are often curious as to whether they have sequenced deep enough to saturate all the binding sites. In principle, sequencing saturation should be dependent on the fold-enrichment, since higher-fold peaks are saturated earlier than lower-fold ones. In addition, due to different cost and throughput considerations, different users might be interested in recovering sites at different fold-enrichment cutoffs. Therefore, MACS produces a saturation table to report, at different fold-enrichments, the proportion of sites that could still be detected when using 90% to 20% of the tags. Such tables produced for FoxA1 (3.9 million tags) and NRSF (2.2 million tags) ChIP-Seq data sets (Figure S4 in Additional data file 1; CTCF does not have a control to robustly estimate fold-enrichment) show that while peaks with over 60-fold enrichment have been saturated, deeper sequencing could still recover more sites less than 40-fold enriched relative to the chromatin input DNA. As sequencing technologies improve their throughput, researchers are gradually increasing their sequencing depth, so this question could be revisited in the future. For now, we leave it up to individual users to make an informed decision on whether to sequence more based on the saturation at different fold-enrichment levels.

The *d *modeled by MACS suggests that some short read sequencers such as Solexa may preferentially sequence shorter fragments in a ChIP-DNA pool. This may contribute to the superior resolution observed in ChIP-Seq data, especially for activating transcription and epigenetic factors in open chromatin. However, for repressive factors targeting relatively compact chromatin, the target regions might be harder to sonicate into the soluble extract. Furthermore, in the resulting ChIP-DNA, the true targets may tend to be longer than the background DNA in open chromatin, making them unfavorable for size-selection and sequencing. This implies that epigenetic markers of closed chromatin may be harder to ChIP, and even harder to ChIP-Seq. To assess this potential bias, examining the histone mark ChIP-Seq results from Mikkelsen *et al*. [7], we find that while the ChIP-Seq efficiency of the active mark H3K4me3 remains high as pluripotent cells differentiate, that of repressive marks H3K27me3 and H3K9me3 becomes lower with differentiation (Table S2 in Additional data file 1), even though it is likely that there are more targets for these repressive marks as cells differentiate. We caution ChIP-Seq users to adopt measures to compensate for this bias when ChIPing repressive marks, such as more vigorous sonication, size-selecting slightly bigger fragments for library preparation, or sonicating the ChIP-DNA further between decrosslinking and library preparation.

MACS calculates the FDR based on the number of peaks from control over ChIP that are called at the same *p*-value cutoff. This FDR estimate is more robust than calculating the FDR from randomizing tags along the genome. However, we notice that when tag counts from ChIP and controls are not balanced, the sample with more tags often gives more peaks even though MACS normalizes the total tag counts between the two samples (Figure S5 in Additional data file 1). While we await more available ChIP-Seq data with deeper coverage to understand and overcome this bias, we suggest to ChIP-Seq users that if they sequence more ChIP tags than controls, the FDR estimate of their ChIP peaks might be overly optimistic.

## Conclusion

As developments in sequencing technology popularize ChIP-Seq, we propose a novel algorithm, MACS, for its data analysis. MACS offers four important utilities for predicting protein-DNA interaction sites from ChIP-Seq. First, MACS improves the spatial resolution of the predicted sites by empirically modeling the distance *d *and shifting tags by *d*/*2*. Second, MACS uses a dynamic λ_local _parameter to capture local biases in the genome and improves the robustness and specificity of the prediction. It is worth noting that in addition to ChIP-Seq, λ_local _can potentially be applied to other high throughput sequencing applications, such as copy number variation and digital gene expression, to capture regional biases and estimate robust fold-enrichment. Third, MACS can be applied to ChIP-Seq experiments without controls, and to those with controls with improved performance. Last but not least, MACS is easy to use and provides detailed information for each peak, such as genome coordinates, *p*-value, FDR, *fold_enrichment*, and *summit *(peak center).

## Materials and methods

### Dataset

ChIP-Seq data for three factors, NRSF, CTCF, and FoxA1, were used in this study. ChIP-chip and ChIP-Seq (2.2 million ChIP and 2.8 million control uniquely mapped reads, simplified as 'tags') data for NRSF in Jurkat T cells were obtained from Gene Expression Omnibus (GSM210637) and Johnson *et al*. [8], respectively. ChIP-Seq (2.9 million ChIP tags) data for CTCF in CD4^+ ^T cells were derived from Barski *et al*. [5].

ChIP-chip data for FoxA1 and controls in MCF7 cells were previously published [1], and their corresponding ChIP-Seq data were generated specifically for this study. Around 3 ng FoxA1 ChIP DNA and 3 ng control DNA were used for library preparation, each consisting of an equimolar mixture of DNA from three independent experiments. Libraries were prepared as described in [8] using a PCR preamplification step and size selection for DNA fragments between 150 and 400 bp. FoxA1 ChIP and control DNA were each sequenced with two lanes by the Illumina/Solexa 1G Genome Analyzer, and yielded 3.9 million and 5.2 million uniquely mapped tags, respectively.

### Software implementation

MACS is implemented in Python and freely available with an open source Artistic License at [16]. It runs from the command line and takes the following parameters: -t for treatment file (ChIP tags, this is the ONLY required parameter for MACS) and -c for control file containing mapped tags; --format for input file format in BED or ELAND (output) format (default BED); --name for name of the run (for example, FoxA1, default NA); --gsize for mappable genome size to calculate λ_BG _from tag count (default 2.7G bp, approximately the mappable human genome size); --tsize for tag size (default 25); --bw for *bandwidth*, which is half of the estimated sonication size (default 300); --pvalue for *p*-value cutoff to call peaks (default 1e-5); --mfold for high-confidence fold-enrichment to find model peaks for MACS modeling (default 32); --diag for generating the table to evaluate sequence saturation (default off).

In addition, the user has the option to shift tags by an arbitrary number (--shiftsize) without the MACS model (--nomodel), to use a global lambda (--nolambda) to call peaks, and to show debugging and warning messages (--verbose). If a user has replicate files for ChIP or control, it is recommended to concatenate all replicates into one input file. The output includes one BED file containing the peak chromosome coordinates, and one xls file containing the genome coordinates, *summit*, *p*-value, *fold_enrichment *and FDR (if control is available) of each peak. For FoxA1 ChIP-Seq in MCF7 cells with 3.9 million and 5.2 million ChIP and control tags, respectively, it takes MACS 15 seconds to model the ChIP-DNA size distribution and less than 3 minutes to detect peaks on a 2 GHz CPU Linux computer with 2 GB of RAM. Figure S6 in Additional data file 1 illustrates the whole process with a flow chart.

## Abbreviations

ChIP, chromatin immunoprecipitation; CTCF, CCCTC-binding factor; FDR, false discovery rate; FoxA1, hepatocyte nuclear factor 3α; MACS, Model-based Analysis of ChIP-Seq data; NRSF, neuron-restrictive silencer factor.

## Authors' contributions

XSL, WL and YZ conceived the project and wrote the paper. YZ, TL and CAM designed the algorithm, performed the research and implemented the software. JE, DSJ, BEB, CN, RMM and MB performed FoxA1 ChIP-Seq experiments and contributed to ideas. All authors read and approved the final manuscript.

## Additional data files

The following additional data are available. Additional data file 1 contains supporting Figures S1-S6, and supporting Tables S1 and S2.

## Supplementary Material

Additional data file 1 Figures S1-S6, and Tables S1 and S2. Click here for file
