# Supplementary material for: Model-based Analysis of ChIP-Seq (MACS)
Source: Genome Biol. 2008 Sep 17;9(9):R137. doi: 10.1186/gb-2008-9-9-r137 (PMC2592715; doi:10.1186/gb-2008-9-9-r137)
Supplement: Additional data file 1 — Figures S1-S6, and Tables S1 and S2. [file gb-2008-9-9-r137-S1.pdf]

Figure S1. Comparison of MACS with other methods on CTCF ChIP-Seq data. (a) CTCF motif occurrence within 50 bp of the peak centers and (b) and average distance from CTCF peak center to motif (peaks with no motif within 150bp from peak center are removed).

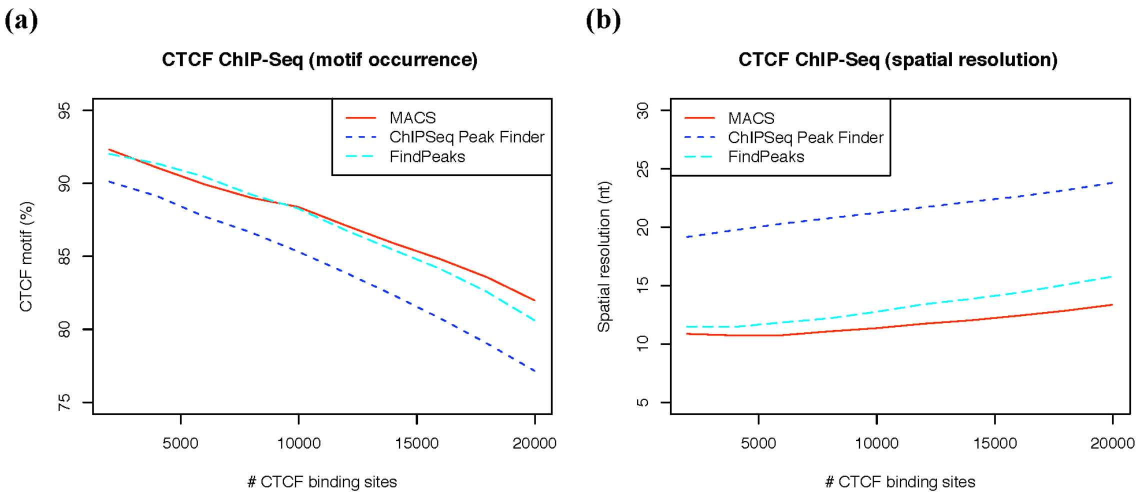

Figure S2. Tag number distributions of FoxA1 ChIP-Seq at ChIP-Seq/chip overlapping peaks, ChIP-chip unique peaks, and genome background. For each peak, the total tag number within the 300 bp region centered at the ChIP-chip peak *summit* is used. Genome background is based on 100,000 randomly selected 300 bp regions in the human genome, excluding regions with ‘N’.

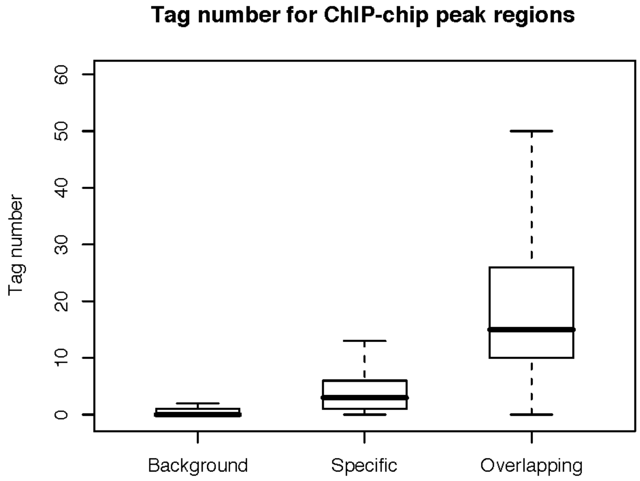

Figure S3. Comparison of NRSF ChIP-Seq and ChIP-chip. (a) Overlap between the NRSF binding sites detected by ChIP-chip (MAT, FDR < 1% and fold enrichment > 2) and ChIP-Seq (MACS, FDR < 1%). Shown are the numbers of regions found to be detected by both platforms (i.e. having at least 1bp in common) or unique to each platform. (b) The distribution of ChIP-Seq tag number and ChIP-chip MATscore for NRSF binding sites identified by both platforms. (c) MATscore distributions of NRSF ChIP-chip at ChIP-Seq/chip overlapping peaks, ChIP-Seq unique peaks, and genome background. For each peak, the mean MATscore for all probes within the 300 bp region centered at the ChIP-Seq peak *summit* is used. Genome background is based on MATscores of all array probes in the NRSF ChIP-chip data. (d) Width distributions of NRSF ChIP-Seq/chip overlapping peaks and ChIP-Seq unique peaks at different fold enrichments (less than 50, from 50 to 100, and larger than 100). (e) Spatial resolution for NRSF ChIP-chip and ChIP-Seq peaks. The Wilcoxon test was used to calculate the p-values for (d) and (e). (f) Motif occurrence (within 2000 bp from *summit*) of NRSF ChIP-Seq/chip overlapping peaks and platform unique peaks. Error bar showing standard deviation is calculated from random sampling ten times of 500 peaks for each category.

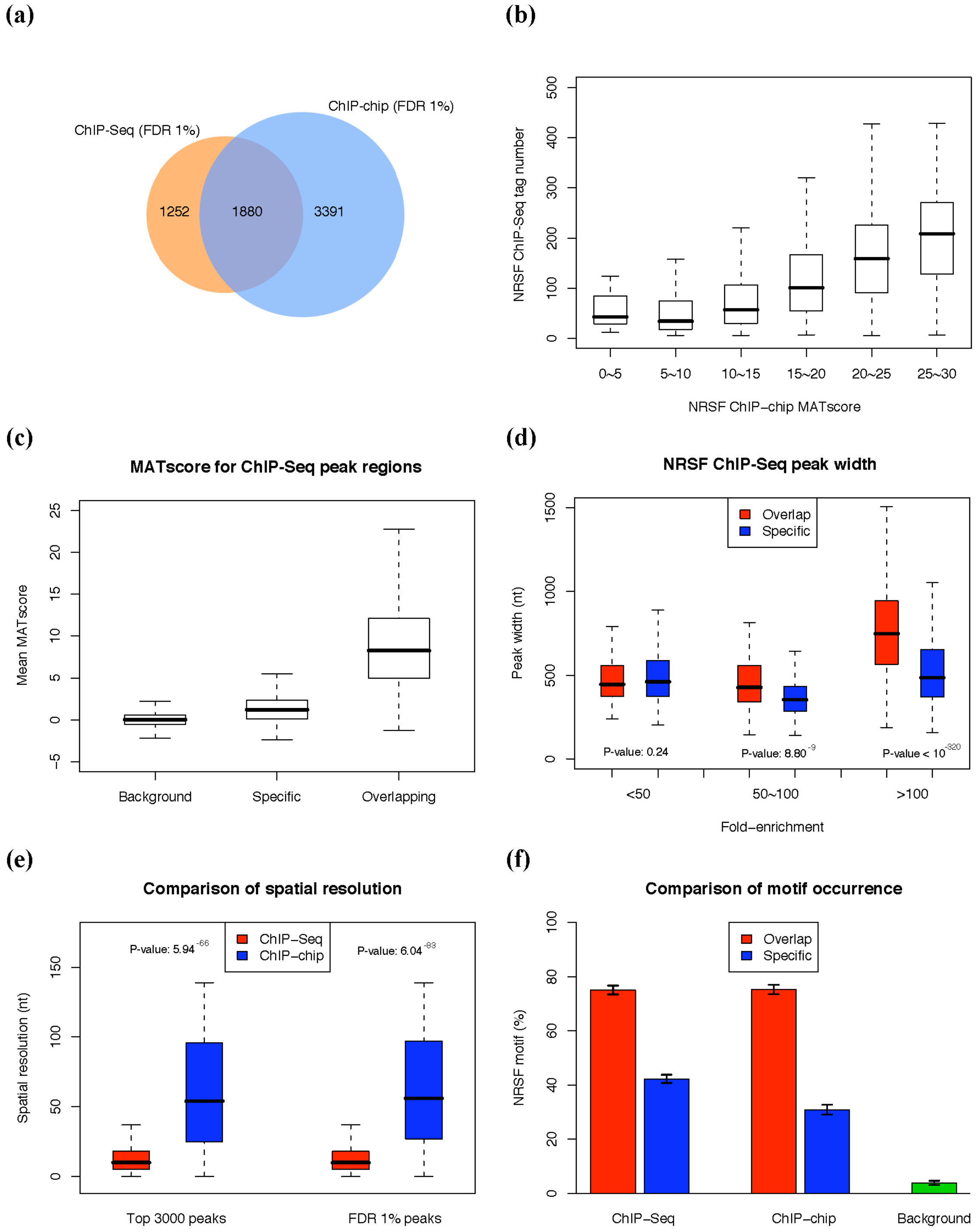
(a)
ChIP-Seq (FDR 1%)
ChIP-chip (FDR 1%)
1252
1880
3391
(b)
NRSF ChIP-Seq tag number
0
100
200
300
400
500
0~5
5~10
10~15
15~20
20~25
25~30
NRSF ChIP−chip MATscore
(c)
MATscore for ChIP-Seq peak regions
Mean MATscore
−5
0
5
10
15
20
25
Background
Specific
Overlapping
(d)
NRSF ChIP-Seq peak width
Peak width (nt)
0
500
1000
1500
Overlap
Specific
P-value: 0.24
P-value: 8.80^{-9}
P-value < 10^{-320}
<50
50~100
>100
Fold−enrichment
(e)
Comparison of spatial resolution
Spatial resolution (nt)
0
50
100
150
ChIP−Seq
ChIP−chip
P-value: 5.94^{-66}
P-value: 6.04^{-83}
Top 3000 peaks
FDR 1% peaks
(f)
Comparison of motif occurrence
NRSF motif (%)
0
20
40
60
80
100
Overlap
Specific
ChIP−Seq
ChIP−chip
Background

Figure S4. Percentage of (a) FoxA1 and (b) NRSF ChIP-Seq peaks (at *p-value* 10^{-5} cutoff) identified from fractions of ChIP-Seq tags. Peaks are categorized according to their fold enrichment. The average results from five separate random sampling of the tags are used to draw plot. The numbers of ChIP-Seq peaks (at *p-value* 10^{-5} cutoff) for different fold enrichment are shown in (c) for FoxA1 ChIP-Seq and (d) for NRSF ChIP-Seq.

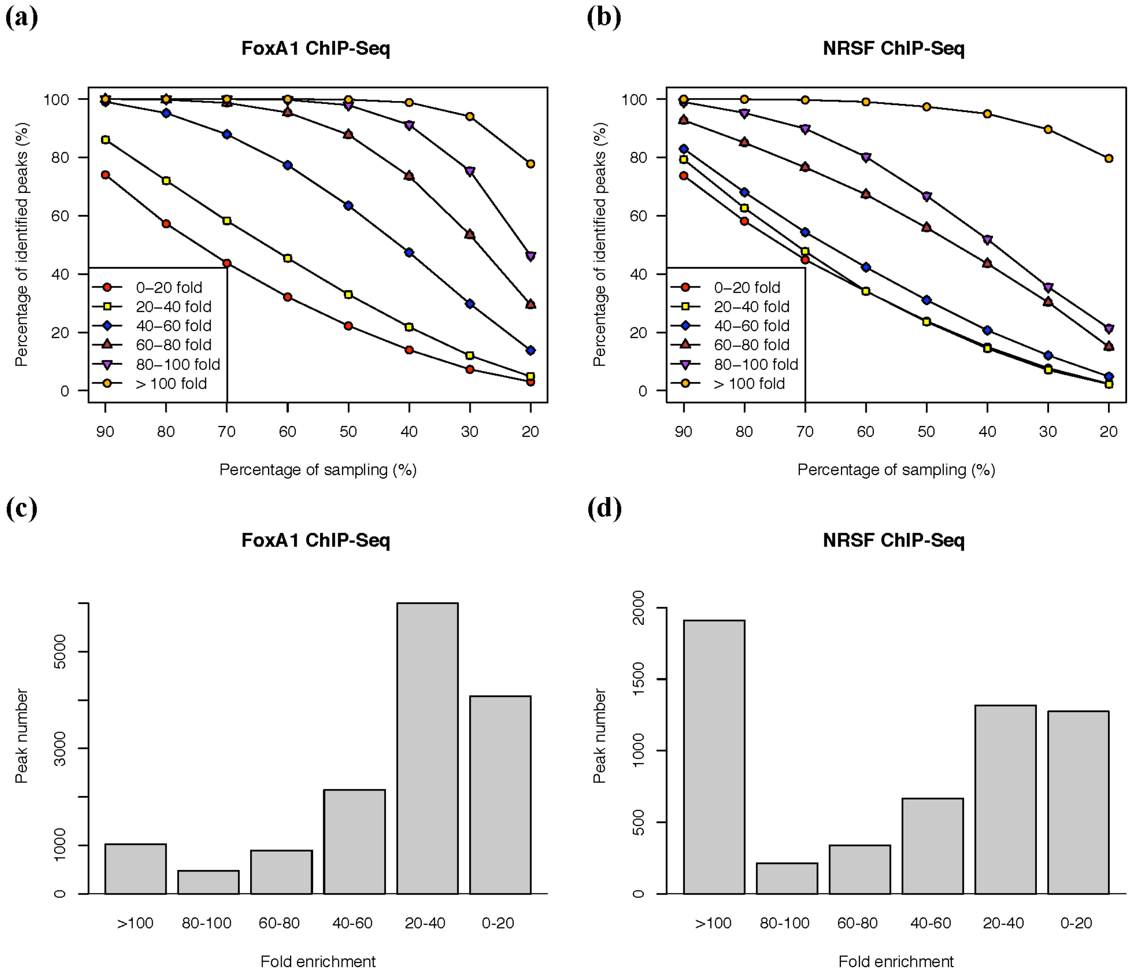

Figure S5. The influence of unbalanced tag numbers between ChIP and control experiments in ChIP-Seq data analysis. With the increase of tag ratio between control and FoxA1 ChIP experiments, (a) to identify the same number of FoxA1 peaks results in higher FDR, and (b) less FoxA1 peaks are identified under the same FDR cutoff. The analysis is based on random sampling of control tags.

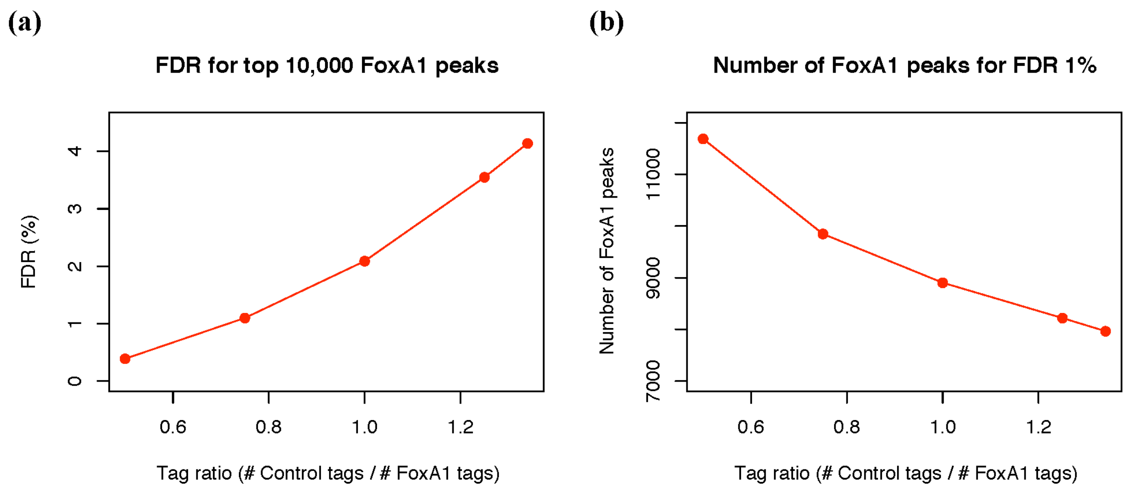

Figure S6. Workflow chart of MACS.

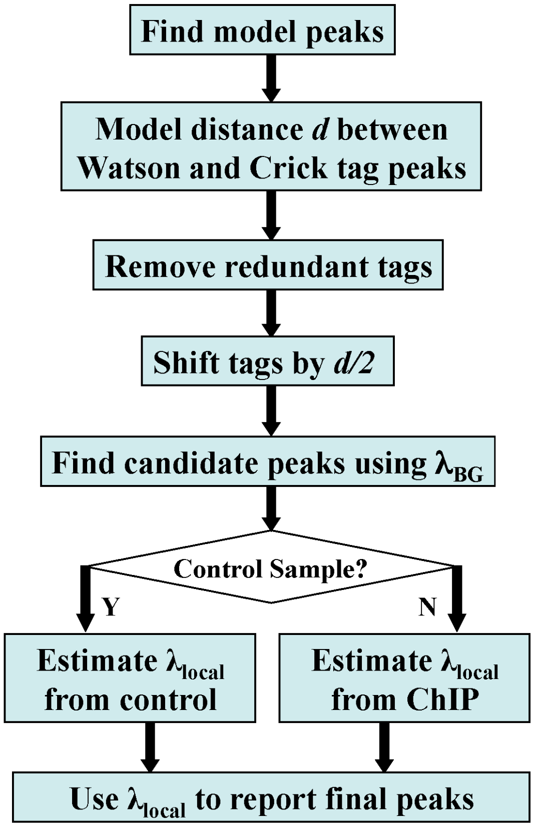

Table S1. Summary of binding sites identified by MACS.

Factor	Cutoff	Number of peaks	Average peak width	% with motif in center 100bp
FoxA1	FDR 1%	7,880	461 bp	58.4 %
NRSF	FDR 1%	3,132	612 bp	53.9 %
CTCF	*p-value* 10^{-5}	27,121	268 bp	75.1 %

Table S2. Number of peaks (reported by Mikkelsen *et al*, Nat 2007) of active histone mark (H3K4me3) and inactive histone marks (H3K27me3 and H3K9me3) as mouse pluriputent cells differentiate (Mikkelsen *et al*, Nat 2007). The first number in each cell is the reported peak number, and the second is the number of sequenced tags.

Peaks / tags	Embryonic stem cells	Embryonic fibroblasts	Neural progenitor cells
H3K4me3	19,524 / 8.9M	16,738 / 11.3M	17,432 / 6.5M
H3K27me3	4,652 / 6.5M	6,548 / 11.4M	2,215 / 7.9M
H3K9me3	1,789 / 4.2M	991 / 3.7M	446 / 3.9M
